# Efficacy and safety of gelatin for fluid therapy in hypovolemia: a systematic review and meta-analysis

**DOI:** 10.1186/cc10415

**Published:** 2011-10-27

**Authors:** CS Hartog, V Vlasakov, DO Thomas-Rueddel, H Rueddel, R Hutagalung, K Reinhart

**Affiliations:** 1 Department of Anesthesiology and Intensive Care Medicine, Jena University Hospital, Jena, Germany; 2 Center for Sepsis Control & Care, Jena University Hospital, Jena, Germany

## Introduction

Gelatin is frequently used as volume expander. There are growing concerns about safety.

## Objective

To systematically assess clinical evidence concerning mortality, coagulation and renal function.

## Methods

Systematic review of randomised controlled trials (RCT) on gelatin in hypovolemia in comparison to any other fluid with a comprehensive search strategy (Ovid Medline (1948 to May 2011), EMBASE (1947 to May 2011), Cochrane Library). Data were independently extracted and risk of bias assessed using the 2010 Cochrane tool. Primary outcome was overall mortality. Secondary outcomes were the number of patients exposed to allogeneic transfusion, frequency of renal replacement therapy (RRT) or acute renal failure (ARF). Albumin and crystalloid solutions were defined as suitable, and other synthetic colloids as unsuitable control fluids since they carry similar risk of side effects. Relative risks (RR) and weighted mean differences with 95% CIs were calculated. Data were pooled using a random-effects model (RevMan 5.1, Cochrane Collaboration).

## Results

The search yielded 1,288 citations, 210 reports were read in full. The final sample contained 72 RCT in English, German, French and Italian, published between 1975 and 2010, with 5,915 patients overall, 2,523 of which received gelatin. The median sample size in the gelatin groups was 20 patients (range 10 to 249). In 53 RCT (74%), the study period was ≤24.0 hours. Total gelatin dose was 20 ml/kg (median, range 6 to 62). Only 38 RCT (53%) used suitable control fluids. Forty-nine RCT (68%) investigated elective surgical patients, mostly from cardiac surgery (32 RCT, 44%). Nine RCT (13%) investigated critically ill patients, six RCT (8%) were in emergency patients and seven RCT (10%) were in children. The RR for mortality was 1.02 (CI 0.87 to 1.19, data from 23 RCT with 2,694 patients which reported mortality). Numbers of patients exposed to allogeneic transfusions were provided in 11 RCT, *n *= 1,148 patients and the RR was 1.16 (0.94 to 1.44). When only studies with suitable control fluids were included, the RR for mortality was 1.13 (0.88 to 1.46, 10 RCT, 1,392 patients) and risk for transfusion exposure was 1.35 (0.88 to 2.08, seven RCT, *n *= 672), tending towards control. Only six RCT (*n *= 662 patients) reported the occurrence of RRT or ARF, five of them in comparison with HES solutions. Three RCT reported anaphylactoid events.

## Conclusion

Most published studies on gelatin are small and short-time, use unsuitable control fluids and report too few events to reliably assess the safety of gelatin.

